# Multisystem fatal metastasis of large cell lung carcinoma (LCLC) masquerading as acute cerebral infarction: A case report

**DOI:** 10.1097/MD.0000000000044224

**Published:** 2025-08-29

**Authors:** Tian-Shui Yu, Bao-Qing Pei, Dong Zhao

**Affiliations:** a Key Laboratory of Evidence Science, China University of Political Science and Law, Ministry of Education, Beijing, China; b School of Biological Science and Medical Engineering, Beihang University, Beijing, China.

**Keywords:** acute cerebral infarction, hematogenous metastasis, large cell lung carcinoma, lung cancer, non-small cell lung carcinoma

## Abstract

**Rationale:**

Large cell lung carcinoma (LCLC) is a rare undifferentiated malignant epithelial tumor of the lung. The diagnostic complexity of LCLC stems from its pronounced histological heterogeneity and diverse clinical presentation, particularly when extrapulmonary manifestations constitute the initial disease presentation, complicating early detection.

**Patient concerns:**

A 58-year-old smoker presented with acute-onset dizziness, lethargy, and communication difficulties lasting 1 day.

**Diagnoses:**

The patient was admitted to the hospital, where extensive hypodense lesions in the right temporal, parietal, and occipital lobes were detected via cranial computed tomography, indicative of acute massive cerebral infarction. Histopathological examination revealed a pulmonary tumor with systemic metastases in the brain, heart, liver, and pancreas. Immunohistochemical staining showed undifferentiated pleomorphic cells that were negative for thyroid transcription factor-1, p40, synaptophysin, and CD56, confirming the WHO-defined null immunophenotype LCLC. Extensive cerebral metastases explained the acute neurological presentation.

**Interventions:**

During the initial phase of the illness, the patient received intravenous edaravone (30 mg/d) and enteric-coated oral aspirin tablets (100 mg daily). On May 15, follow-up magnetic resonance imaging revealed acute multifocal infarcts involving the bilateral frontal–parietal lobes and the right temporo-occipital regions. This radiographic progression prompted escalation of therapy, including edaravone intensification, neuroprotection (cerebroprotein hydrolysate), and supportive care (intravenous infusion of 250 mL of 5% glucose solution containing 2 g calcium gluconate and 3 g vitamin C).

**Outcomes:**

The patient succumbed following a 2-month hospitalization during which acute cerebral infarction was the primary treatment focus.

**Lessons:**

This case highlights LCLC’s diagnostic complexity, aggressive metastasis, and potential for non-respiratory initial manifestations of LCLC. Early whole-body imaging and multidisciplinary evaluation are imperative to detect occult malignancies in unexplained stroke cases.

## 1. Introduction

Lung cancer is the principal cause of cancer-related mortality worldwide,^[1]^ with histological classification of tumors into small cell lung carcinoma and non-small cell lung carcinoma (NSCLC).^[2]^ NSCLC is further categorized into 3 principal subtypes: adenocarcinoma, squamous cell carcinoma, and large cell lung carcinoma (LCLC).^[3]^ Over the past decade, the convergence of pathological, genomic, and clinical advancements has driven the systematic reclassification of LCLC into distinct molecular pathological entities.^[4]^ This evolution is codified in the 2021 WHO Classification of Thoracic Tumors, which defines pulmonary LCLC as a rare (<1% of NSCLCs), undifferentiated epithelial malignancy demonstrating negative immunohistochemical evidence of adenocarcinoma, squamous cell carcinoma, and neuroendocrine differentiation, while excluding small cell carcinoma cytomorphology (nuclear molding and scant cytoplasm).^[5]^ Thus, LCLC constitutes a definitive diagnostic designation of exclusion in surgically resected NSCLC specimens.

The diagnostic challenges of LCLC arise from its marked histomorphological diversity and nonspecific clinical presentation. Notably, extrapulmonary manifestations, such as the initial disease phenotype, frequently delay the definitive diagnosis. This clinicopathological analysis describes a rare lethal LCLC case masquerading as an acute cerebral infarction, integrating a systematic review of its clinical performance, histopathological characteristics, and multidisciplinary diagnostic dilemmas.

## 2. Case report

**Figure 1.**
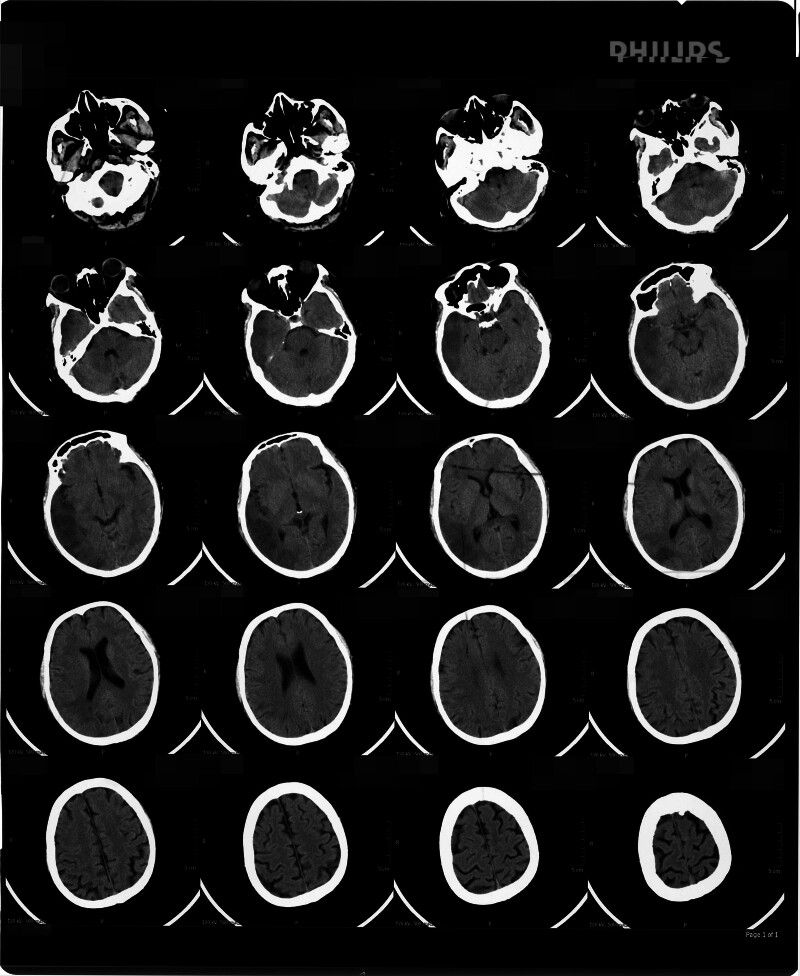
Cranial CT imaging demonstrated an extensive hypodense lesion involving the right temporo-parieto-occipital region on May 13, radiologically characteristic of acute cerebral infarction. CT = computed tomography.

**Figure 2.**
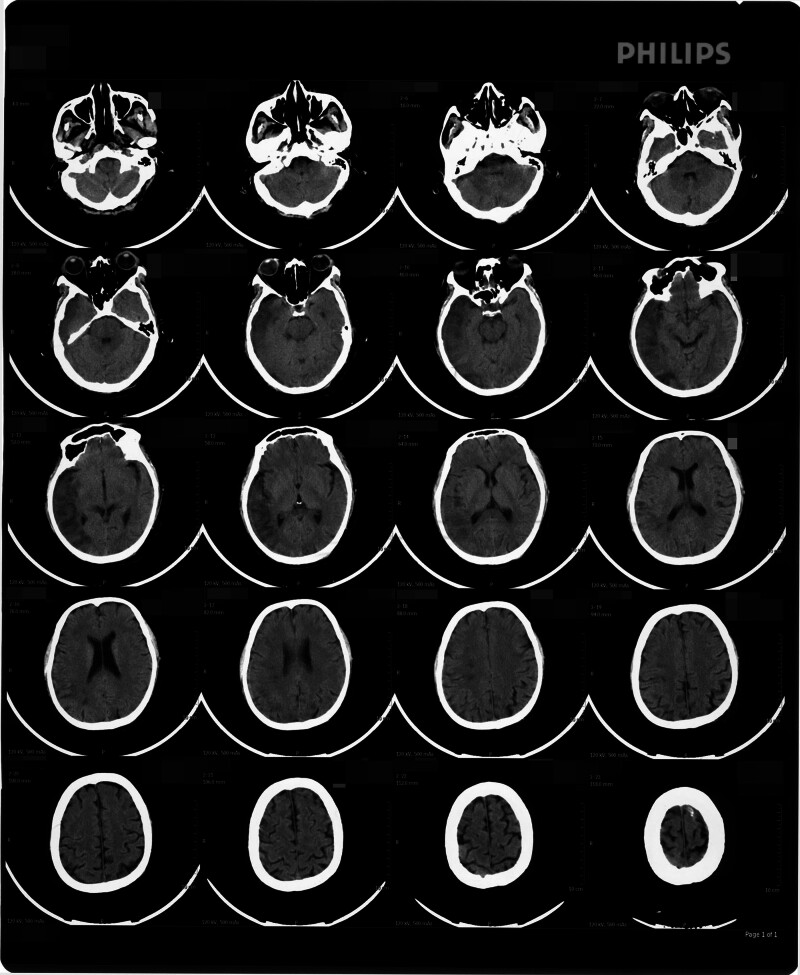
Cranial computed tomography imaging on May 22 showed improvement of the cerebral infarction compared with the results from May 13.

**Figure 3.**
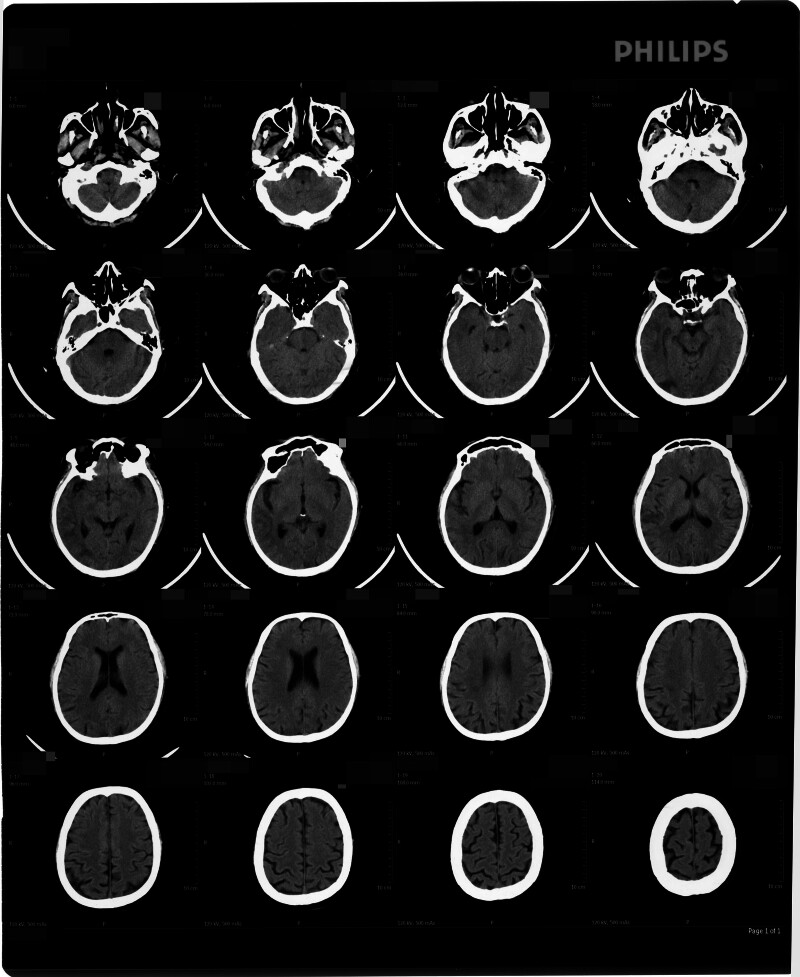
Cranial computed tomography imaging on May 27 showed improvement of the cerebral infarction compared with the results from May 13 and May 22.

**Figure 4.**
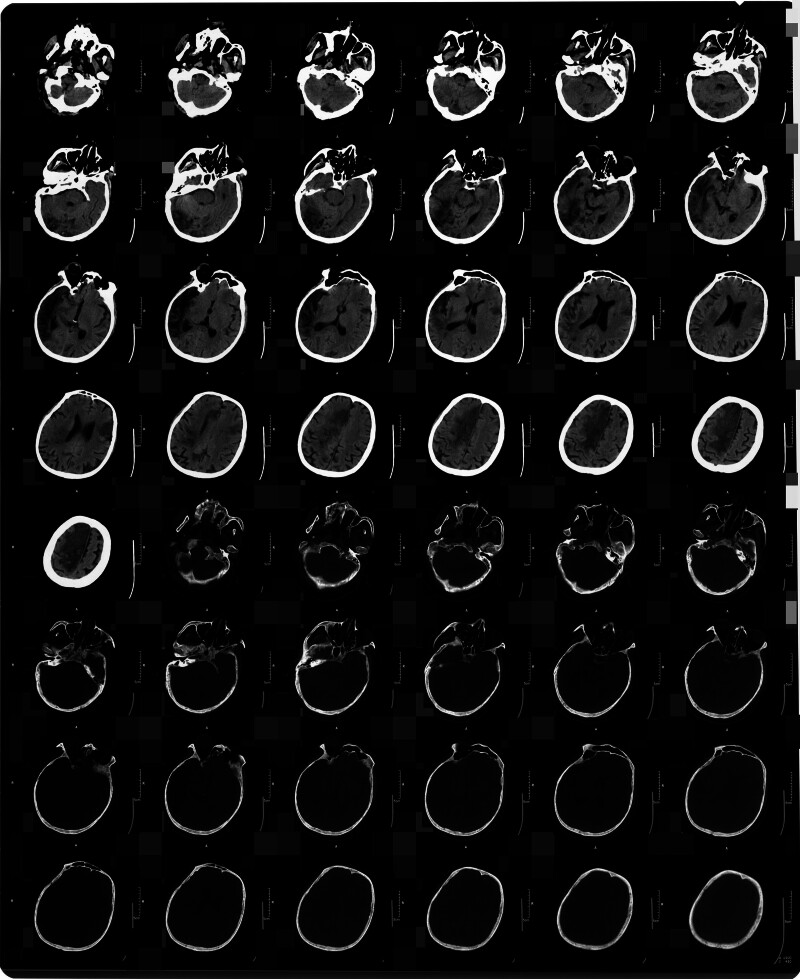
Cranial CT imaging demonstrated irregular hypoattenuating lesions in the right temporoparietal region and bilateral fronto-occipital areas on July 3. CT = computed tomography.

On May 13, 2023, a 58-year-old Han Chinese male with a smoking history was hospitalized because of acute-onset dizziness, lethargy, and communication difficulties lasting 1 day. The patient denied a personal history of congenital genetic disorders or familial predisposition to neoplastic disorders. Cranial computed tomography (CT) on admission revealed extensive hypodense lesions in the right temporal, parietal, and occipital lobes indicative of an acute massive cerebral infarction (Fig. 1). Immediate interventions included intravenous edaravone (30 mg) and enteric-coated oral aspirin tablets (100 mg). By May 14, neurological examination demonstrated sluggish responsiveness, limited verbal output, and left upper limb numbness, with preserved lower limb motor function. Subsequent magnetic resonance imaging on May 15 confirmed acute multifocal infarcts involving the bilateral frontoparietal lobes and right temporo-occipital regions, prompting intensified therapy with edaravone, cerebroprotein hydrolysate, and intravenous fluids (250 mL of 5% glucose + 2 g calcium gluconate + 3 g vitamin C). Follow-up cranial CT studies obtained on May 22 and 27 demonstrated right temporo-occipital infarction, as documented in Figures 2 and 3. On July 2, 2023, blurred vision emerged, and ophthalmologic evaluation suggested poststroke homonymous hemianopia. The patient’s transfer to rehabilitation was preceded by cranial and chest CT findings on July 3 demonstrating: Irregular hypoattenuating lesions in the right temporoparietal region and bilateral fronto-occipital areas, consistent with bilateral cerebral infarction and evolving encephalomalacia (Fig. 4); Bilateral pulmonary ground-glass opacities, considered as bilateral pulmonary edema; Concomitant pleural and pericardial effusions. At 09:00 on July 6, the patient presented with fever (peak temperature 38.5°C) and nonproductive cough. Physical examination revealed agitation and noncooperation. Bilateral lung auscultation showed coarse breath sounds with abundant coarse rales. At 11:50, the patient developed altered mental status (lethargy/drowsiness), persistent fever, and respiratory distress with a respiratory rate of 41 breaths per minute. At 18:30, the patient’s consciousness disturbance and respiratory distress progressively worsened. The patient was now in a state of light coma (semicoma). At 09:00 on July 7, the patient presented with a body temperature of 38.0°C and impaired consciousness. Tachypnea was noted on observation. Auscultation of the lungs revealed bilateral coarse breath sounds. At 09:00 on July 8, vital signs were within normal limits for temperature, but the patient exhibited marked tachypnea with a respiratory rate of 49 to 50 breaths per minute and a heart rate of 120 to 123 beats per minute. Positioning changes and chest physiotherapy (including percussion and postural drainage) were initiated to facilitate sputum expectoration. Electrocardiogram revealed sinus tachycardia and nonspecific ST-T segment abnormalities. At 09:00 on July 9, the patient presented with marked agitation and tachypnea (respiratory rate: 43 breaths per minute), requiring supplemental oxygen via face mask. Continuous monitoring revealed tachycardia (heart rate: 133 beats per minute), blood pressure of 125/85 mm Hg, and hypoxemia (oxygen saturation: 81%). Impaired consciousness was noted, accompanied by multiple ecchymoses diffusely distributed over the body. Pulmonary auscultation demonstrated coarse breath sounds with audible gurgling rhonchi bilaterally, indicating retained airway secretions. At 09:00 on July 10, the patient presented critically ill, exhibiting marked tachypnea with labored mouth breathing. Continuous vital sign monitoring revealed: Heart rate 143 beats per minute, blood pressure 129/92 mm Hg, oxygen saturation 68%, and respiratory rate 52 breaths per minute. At 10:01 on July 10, cardiopulmonary arrest ensued, with undetectable blood pressure, absent pulses, fixed dilated pupils (8 mm), and loss of brainstem reflexes. Despite advanced resuscitation, asystole persisted, and death was declared at 10:46.

**Figure 5.**
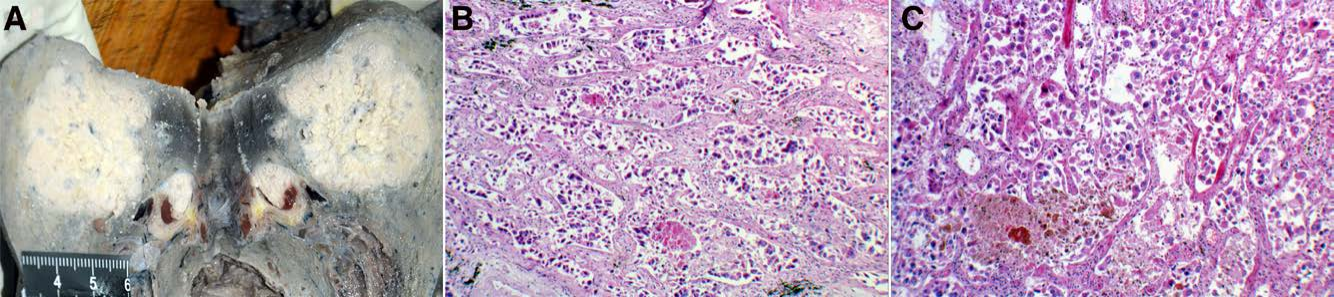
Gross pathological and histopathological observations. (A) The resected right lower lobe specimen contained a poorly circumscribed tumor with gray-white cut surfaces and tumor invasion into the bronchial lumen at the right pulmonary hilum. (B, C) Neoplastic cells in the right lower lobe of the lung were composed of medium-to-large sized cells with irregular nuclei and frequent atypical mitoses.

**Figure 6.**
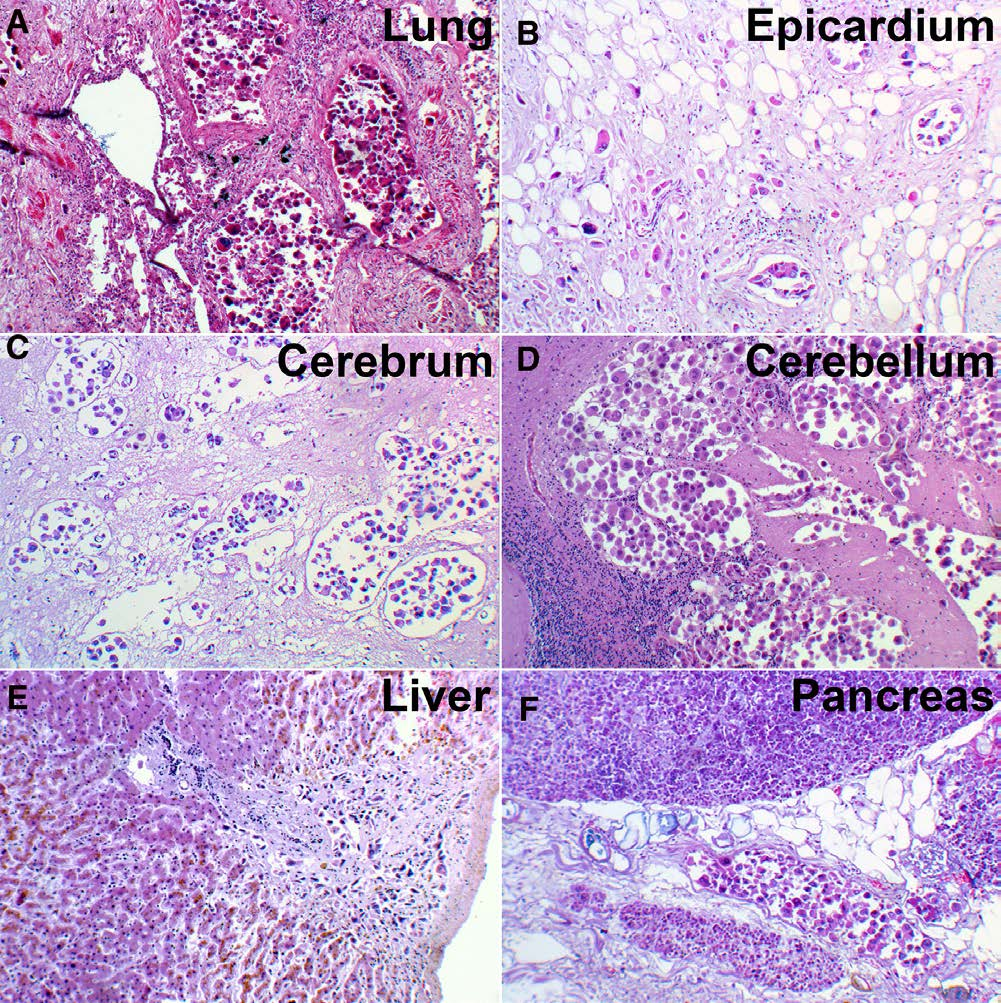
Histopathological findings in multiple organs. (A) Neoplastic cells were identified within the vasculature of the pulmonary interstitium. (B) Neoplastic cells were identified within the epicardial vasculature, accompanied by extensive neoplastic infiltration into the perivascular connective tissue. (C, D) Numerous neoplastic cells were identified within the vasculature of the cerebral white matter and cerebellar cortex, accompanied by neoplastic infiltration into the adjacent brain parenchyma. (E) Neoplastic cells were identified within the portal tracts of the liver. (F) Neoplastic cells are identified within the vasculature of the pancreatic capsule.

**Figure 7.**
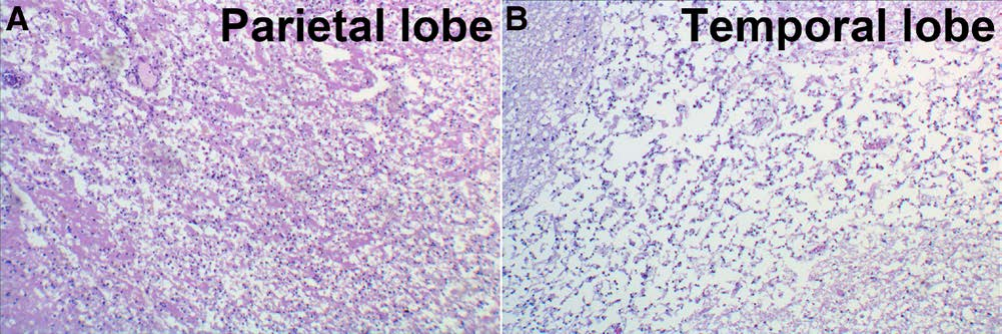
Extensive cerebral infarction in the right parietal lobe (A) and right temporal lobe (B), with the right temporal lobe being more prominently involved (B).

An autopsy was performed 3 days after death to ascertain the cause of death. A poorly circumscribed, unencapsulated tumor (4.7 × 4.0 cm) was identified in the right lower lobe adjacent to the pulmonary hilum, demonstrating invasive growth into the main bronchus (Fig. 5A). The lesion exhibited a homogeneous grayish-white cut surface with infiltrative margins in the surrounding pulmonary parenchyma (Fig. 5A). Microscopic examination revealed that the tumor was composed of pleomorphic cells arranged in nodules ranging from medium to large in size, with irregular nuclear contours and atypical mitoses, and demonstrated no glandular, squamous, or neuroendocrine differentiation (Fig. 5B, C). Systemic tumor embolization demonstrated multifocal involvement of the pulmonary, cardiac, cerebral, hepatic, and pancreatic vasculature, with concurrent local neoplastic infiltration into the adjacent tissues (Fig. 6A–F). Multiple patchy necrotic foci with microglial infiltration were present in the right temporal and bilateral parietal/occipital lobes (Fig. 7A, B).

**Figure 8.**
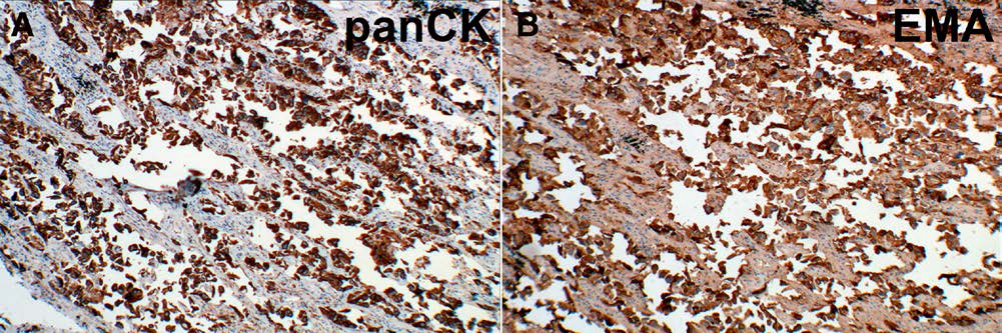
Immunohistochemical findings of neoplastic cells in the right lower lobe of the lung. The tumor cells exhibited diffuse cytoplasmic positivity for panCK and EMA, with immunoreactivity patterns illustrated in A, B. EMA = epithelial membrane antigen, panCK = pancytokeratin.

The tumor cells demonstrated cytoplasmic immunoreactivity for pancytokeratin (Fig. 8A) and epithelial membrane antigen (Fig. 8B), but were negative for thyroid transcription factor-1 (TTF-1), carcinoembryonic antigen, CK5/6, p40, p63, leukocyte common antigen, synaptophysin, and CD20/30/56.

## 3. Discussion

The 2004 WHO classification categorized LCLC as a diagnostic entity encompassing variants, including large cell neuroendocrine carcinoma (LCNEC), basaloid carcinoma, lymphoepithelioma-like carcinoma, clear cell carcinoma, and rhabdoid phenotype carcinoma, with no requirement for immunohistochemical (IHC) evaluation of adenocarcinoma (e.g., TTF-1) or squamous (e.g., p40) markers.^[6]^ In contrast, the 2015 WHO classification mandates IHC-driven reclassification of solid-pattern carcinomas: TTF-1-positive tumors are designated solid adenocarcinoma, while p40-positive tumors are classified as non-keratinizing squamous cell carcinomas, reflecting molecular evidence that historical “large cell carcinomas” represent heterogeneous lineages or immunophenotypically null tumors.^[7]^ This reclassification was supported by molecular and IHC evidence demonstrating that previously defined LCLC comprised heterogeneous entities with distinct differentiation lineages (adenocarcinoma, squamous, and neuroendocrine) or a null immunophenotype and genotype.^[8–10]^ Poorly differentiated neoplasms demonstrating absent expression of lineage-specific immunohistochemical markers are classified as null phenotypes and are characterized by negative results for TTF-1, p40, synaptophysin, and CD56. Furthermore, epidemiological evidence from the National Cancer Institute Surveillance Epidemiology and End Results registry revealed that a marked decline in LCLC diagnoses coinciding with the clinical adoption of TTF-1 immunohistochemistry–demonstrating a diagnostic shift as pathologists progressively reclassified these tumors into lineage-specific subtypes (e.g., adenocarcinoma and squamous) using modern biomarkers.^[11]^ In this case, the tumor cells were negative for TTF-1, p40, synaptophysin, and CD56, combined with the absence of glandular, squamous, or neuroendocrine differentiation, supporting the pathological diagnosis of LCLC with a “null immunophenotype.”

LCLC cases lacking definitive immunophenotypes require genomic profiling to identify targetable oncogenic pathways that are shared with conventional NSCLC subtypes. Persistent nomenclature challenges arise from histopathological diagnosis preceding the genomic analysis. Thus, LCLC should be considered a provisional diagnosis, modifiable to “LCLC-genetically ADC/SQC,” upon identifying lineage-specific molecular profiles. Advancing genomic and expression analytics is likely to resolve classification ambiguities, potentially rendering the LCLC category obsolete. A study of 102 cases of LCLC revealed that adenocarcinoma-associated mutations (EGFR, KRAS, BRAF, and ALK) occurred exclusively in the null immunophenotype or adenocarcinoma-differentiated LCLC. KRAS mutations were more frequent in the null immunophenotype (>40%), whereas EGFR mutations predominated in adenocarcinoma phenotypes.^[8,9]^ Most LCLC cases were reclassified as adenocarcinomas or squamous carcinomas after immunohistochemical subtyping, with rare residual null or indeterminate immunophenotypes. A subset of null immunophenotype cases shows adenocarcinoma-like genetic changes (KRAS and occasional EGFR mutations).^[12]^ Molecular testing-guided targeted therapy is recommended for null immunophenotypes. The patient’s death prior to definitive diagnosis precluded the execution of comprehensive molecular profiling during the hospitalization period.

The current standard treatment for LCLC primarily involves surgical resection while the necessity of postoperative chemotherapy remains controversial.^[13,14]^ Although biological therapies have shown efficacy in various solid tumors,^[15]^ their effectiveness in LCLC remains unverified. A follow-up study of 83 patients with LCLC revealed a significantly higher 1-year survival rate in radical resection patients (65.1%) than in palliative treatment recipients (35.0%), with no significant survival difference between the postoperative chemoradiotherapy and surgery alone groups.^[16]^ Studies from Japan have also demonstrated significantly improved 5-year survival rates in surgically treated LCLC patients compared with nonsurgical cases.^[13]^ Furthermore, evidence suggests that multimodal therapeutic strategies yield better long-term outcomes than surgery alone,^[17]^ although these conclusions may require cautious interpretation due to potential limitations in the follow-up duration.

In addition to surgical management, the prognostic outcomes of LCLC demonstrate significant dependence on the tumor–node–metastasis classification established during the initial diagnostic evaluation. Tumor–node–metastasis staging (including N and M classifications) significantly correlated with 1-year survival in 83 patients with LCLC.^[16]^ Survival rates decreased progressively with advancing stages: stage I (highest) > II > III > IV, N0 > N1/N2/N3, and M0 > M1.^[16]^ Clinically, LCLC manifests insidiously with nonspecific symptoms, often resulting in delayed diagnosis until the advanced stages. LCLC exhibits a more aggressive biological behavior than other NSCLC variants. The neoplasm demonstrates aggressive biological behavior, characterized by rapid progression and large primary lesions at initial detection. Notably, most reported cases of colony-stimulating factor-producing lung malignancies are histologically classified as LCLC, with colony-stimulating factor hypothesized to potentiate tumor proliferation and metastatic competence. Approximately one-third of the patients with LCLC present with synchronous distant metastases at diagnosis. Predominant metastatic sites include the brain, liver, bone, and distant lymph node.^[18,19]^ Herein, we report a rare case of highly aggressive LCLC. The patient succumbed within weeks following the initial acute cerebral infarction, with extensive hematogenous metastases to multiple organs (including the brain, heart, liver, and pancreas) confirmed only at autopsy. Autopsy further revealed that the extent and number of metastatic tumors in the brain were more pronounced than those in other organs, thereby corroborating the etiology of the patient’s initial presentation of acute cerebral infarction.

This case highlights acute cerebral infarction as a critical initial manifestation of LCLC, emphasizing its role in masking underlying malignancy. Clinicians should maintain heightened suspicion of occult neoplasms when evaluating cryptogenic stroke, particularly in patients with atypical neurological deficits or rapid clinical deterioration. Early whole-body imaging and multidisciplinary evaluation are imperative to detect occult malignancies in unexplained stroke cases. This case reinforces the LCLC’s position as a diagnosis of exclusion and a critical target for advancements in precision oncology.

## Author contributions

**Investigation:** Tian-Shui Yu, Bao-Qing Pei.

**Methodology:** Tian-Shui Yu, Dong Zhao.

**Supervision:** Bao-Qing Pei, Dong Zhao.

**Validation:** Bao-Qing Pei, Dong Zhao.

**Writing – original draft:** Tian-Shui Yu, Bao-Qing Pei.

**Writing – review & editing:** Tian-Shui Yu, Bao-Qing Pei, Dong Zhao.
